# Outcome risk model development for heterogeneity of treatment effect analyses: a comparison of non-parametric machine learning methods and semi-parametric statistical methods

**DOI:** 10.1186/s12874-024-02265-8

**Published:** 2024-07-23

**Authors:** Edward Xu, Joseph Vanghelof, Yiyang Wang, Anisha Patel, Jacob Furst, Daniela Stan Raicu, Johannes Tobias Neumann, Rory Wolfe, Caroline X. Gao, John J. McNeil, Raj C. Shah, Roselyne Tchoua

**Affiliations:** 1 Jarvis College of Computing and Digital Media, DePaul University, Chicago, IL United States of America; 2 Rush Alzheimer’s Disease Center, Rush University Medical Center, Chicago, IL United States of America; 3 Department of Family & Preventive Medicine, Rush University Medical Center, Chicago, IL United States of America; 4 Department of Cardiology, University Heart & Vascular Centre Hamburg, Hamburg, Germany; 5 German Centre for Cardiovascular Research (DZHK), Partner Site Hamburg/Kiel/Lübeck, Hamburg, Germany; 6 Department of Epidemiology and Preventive Medicine, School of Public Health and Preventive Medicine, Monash University, Melbourne, VIC Australia; 7 Monash University Clinical Trials Centre, Monash University, Melbourne, VIC Australia; 8 Centre for Youth Mental Health, University of Melbourne, Parkview, VIC Australia; 9 Orygen, Parkview, VIC Australia

**Keywords:** Heterogeneity of treatment effect, Random forest, Decision tree, Aspirin, Outcome risk modelling, Disability-free longevity, Clinical trial

## Abstract

**Background:**

In randomized clinical trials, treatment effects may vary, and this possibility is referred to as heterogeneity of treatment effect (HTE). One way to quantify HTE is to partition participants into subgroups based on individual’s risk of experiencing an outcome, then measuring treatment effect by subgroup. Given the limited availability of externally validated outcome risk prediction models, internal models (created using the same dataset in which heterogeneity of treatment analyses also will be performed) are commonly developed for subgroup identification. We aim to compare different methods for generating internally developed outcome risk prediction models for subject partitioning in HTE analysis.

**Methods:**

Three approaches were selected for generating subgroups for the 2,441 participants from the United States enrolled in the ASPirin in Reducing Events in the Elderly (ASPREE) randomized controlled trial. An extant proportional hazards-based outcomes predictive risk model developed on the overall ASPREE cohort of 19,114 participants was identified and was used to partition United States’ participants by risk of experiencing a composite outcome of death, dementia, or persistent physical disability. Next, two supervised non-parametric machine learning outcome classifiers, decision trees and random forests, were used to develop multivariable risk prediction models and partition participants into subgroups with varied risks of experiencing the composite outcome. Then, we assessed how the partitioning from the proportional hazard model compared to those generated by the machine learning models in an HTE analysis of the 5-year absolute risk reduction (ARR) and hazard ratio for aspirin vs. placebo in each subgroup. Cochran’s Q test was used to detect if ARR varied significantly by subgroup.

**Results:**

The proportional hazard model was used to generate 5 subgroups using the quintiles of the estimated risk scores; the decision tree model was used to generate 6 subgroups (6 automatically determined tree leaves); and the random forest model was used to generate 5 subgroups using the quintiles of the prediction probability as risk scores. Using the semi-parametric proportional hazards model, the ARR at 5 years was 15.1% (95% CI 4.0–26.3%) for participants with the highest 20% of predicted risk. Using the random forest model, the ARR at 5 years was 13.7% (95% CI 3.1–24.4%) for participants with the highest 20% of predicted risk. The highest outcome risk group in the decision tree model also exhibited a risk reduction, but the confidence interval was wider (5-year ARR = 17.0%, 95% CI= -5.4–39.4%). Cochran’s Q test indicated ARR varied significantly only by subgroups created using the proportional hazards model. The hazard ratio for aspirin vs. placebo therapy did not significantly vary by subgroup in any of the models. The highest risk groups for the proportional hazards model and random forest model contained 230 participants each, while the highest risk group in the decision tree model contained 41 participants.

**Conclusions:**

The choice of technique for internally developed models for outcome risk subgroups influences HTE analyses. The rationale for the use of a particular subgroup determination model in HTE analyses needs to be explicitly defined based on desired levels of explainability (with features importance), uncertainty of prediction, chances of overfitting, and assumptions regarding the underlying data structure. Replication of these analyses using data from other mid-size clinical trials may help to establish guidance for selecting an outcomes risk prediction modelling technique for HTE analyses.

**Supplementary Information:**

The online version contains supplementary material available at 10.1186/s12874-024-02265-8.

## Background

By design, randomized clinical trials (RCTs) provide information about the average treatment effect for an intervention. Heterogeneity of treatment effect (HTE) refers to the circumstance in which treatment outcomes vary within a population. For example, in an RCT, it may be the case that certain types of participants experience a large decrease in mortality, while the majority experience a modest increase in mortality. In that study, the average treatment effect would indicate a moderate decrease in mortality, but would be poorly representative of the experiences of participants. The discrepancy in treatment effect is a defining characteristic of HTE.

One traditional way to assess HTE in RCTs is to conduct “one-variable-at-a-time” subgroup analyses, evaluating whether treatment effect differs across demographics or baseline risk factors [1]. This approach has been criticized for its tendency to produce false positives due to the many tests performed, false negatives when subgroups are limited in size, and limited clinical applicability since research participants have many traits that simultaneously influence outcomes [2].

A proposed alternative to conventional subgroup analysis is to create subgroups based on research participants’ baseline predicted risk of experiencing an event [3]. In this framework, investigators (1) identify an externally validated predictive risk model for the primary outcome of interest; (2) compute the predicted risk for each participant; (3) partition the participants into subgroups based on the predicted risk; and (4) test for HTE by subgroup. [3]. This approach offers an opportunity to demonstrate HTE in the circumstance that treatment outcomes are correlated with the baseline risk of experiencing that outcome.

Investigators assessing HTE with this approach may discover there are limited externally derived risk prediction tools applicable to their study population or outcome of interest. In such cases, prediction tools may be developed using data from the cohort they are investigating (i.e., an internally developed outcome risk prediction model) [3]. For internal models, development and accuracy are dependent on the number of samples and the frequency of the outcome of interest. Such models must be fine-tuned to prevent overfitting. Traditionally, predictive risk models have been developed using logistic regression, a fully parametric approach, or Cox proportional hazard regression, a semi-parametric approach. However, these models require assumptions of the underlying data structure and significant expert clinical knowledge.

More recently, supervised machine learning models, such as random forests and decision trees, have shown utility towards the development of predictive risk models. Both approaches are ubiquitous, nonparametric (i.e., they make no assumptions about the data distribution), and explainable predictive models that can provide insights into feature importance with respect to predicted outcomes. For example, decision trees can be translated into human-readable “if-then” rules. These approaches also produce partitions in data which maximize the homogeneity with respect to the predicted outcome. For example, decision trees use a partition-based algorithm which separates subjects into homogeneous subgroups with respect to the outcome. The random forest is an ensemble of decision tree designed to reduce variability by aggregating results from multiple decision trees. Whether supervised learning models perform comparably to semi-parametric models for partitioning participants on HTE modelling has not been fully explored.

The ASPirin in Reducing Events in the Elderly (ASPREE) study (Clinical trial registry number: NCT01038583) was a double-blind, randomized controlled trial that assigned participants to aspirin 100 mg daily or placebo starting in 2010 [4]. A total of 19,114 study participants were recruited from Australia and the United States (US). Of the total, 2,411 participants were from the US. Participants must have been at least 70 years of age, or at least 65 years of age if African American or Hispanic in the US, and free of diagnoses of cardiovascular disease, dementia, or physical disability. The primary outcome was a composite of death, dementia, or persistent physical disability. We will refer to this as disability-free longevity. The overall finding of the ASPREE study was that daily low-dose aspirin conferred neither benefit nor harm on the primary outcome (HR = 1.01 95% CI: 0.92–1.11 *p* = 0.79) [5]. A conventional subgroup analysis was conducted for 12 pre-specified measures. Treatment benefit had statistically significant variation by frailty, but by none of the other measures [5].

After publication of the main ASPREE findings described above, a semi-parametric model for risk prediction was developed with the overall study data and published [6]. This work provided an opportunity to examine the properties of the predictive risk model in HTE analyses to use as a standard for determining if partition-based supervised machine learning models (decision trees and random forests) yielded comparable HTE conclusions on the absolute and relative scales. We were interested in partition model performance using a medium sized dataset reflecting a typical clinical trial [7].

## Methods

To conduct the comparative analyses of outcome risk models, we divided our process into four steps: (1) data preparation; (2) models for generating subgroups; (3) assessment of model predictive ability; and (4) model performance in heterogeneity of treatment effect analyses.

### Data preparation

**Fig. 1:**
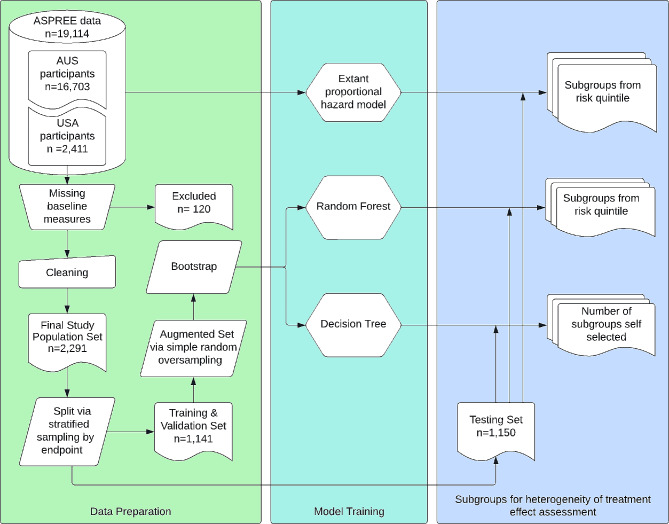
Participant flow diagram and methodology overview

As shown in Fig. 1, US participants who did not have any missing features were selected from the ASPREE dataset. The entire dataset was used for the analyses for the extant, semi-parametric, proportional hazards predictive risk model. The cohort was then split 50%/50% into two sets: (1) a training and validation set, which was used to develop the machine learning models; and (2) a testing set, used for assessing model performance.

We used a stratified sampling approach to ensure the sets retained a similar ratio of the composite outcome. In ASPREE, only about 10% of participants experienced the outcome by the end of the study. Machine learning techniques tend to learn more about the outcome type for which they have more examples. This can result in models which have poor sensitivity for underrepresented outcome types yet exhibit high overall accuracy. To address this potential for biased learning, we created an augmented training and validation set by randomly oversampling participants who experienced the outcome with replacement until the count matched that of the participants with disability free longevity. The test set was not altered and was representative of the original participant population (10% who had the composite outcome).

### Models for generating subgroups

Three approaches for generating subgroups were selected: (1) a proportional hazards model; (2) a decision tree model; and (3) a random forest model. The outcome for all models was a composite of experiencing death, dementia, or persistent physical disability. The proportional hazards model accounted for time to the event and censoring, while the machine learning models accounted solely for whether the event occurred or not.

#### Extant semi-parametric proportional hazards predictive risk model:

A literature search was conducted to identify published models predicting the primary composite outcome or individual components. Neumann et al. used Cox proportional hazard regression to predict the 5-year risk of the primary composite endpoint in ASPREE [6]. Proportional hazards regressions are semi-parametric, time-to-event models; a non-parametric component specifies a baseline hazard function; and a parametric portion specifies how the log of the hazard function varies linearly with the covariates. The authors selected 24 baseline measures as candidate predictors in their analysis [6], indicated in Appendix 1. The candidate features to were used to create two models, one for men and one for women, Appendix 2. To create subgroups for assessing HTE, the sex-specific models were used to generate a risk prediction score for each US ASPREE participant with non-missing data. Then, participants were stratified into subgroups by risk quintile, with group 1 containing the fifth with lowest predicted risk, and group 5 containing the fifth with highest predicted risk.

### Supervised non-parametric machine learning outcome classifiers

While supervised models are classically used to predict outcomes, we used them for subgrouping, based on outcome. As such, while we tuned our models to prevent overfitting, we focused on generating stable subgroups rather than sensitivity analysis and optimization of accuracy. As shown in Appendix 1, the machine learning models were developed using a total of 26 baseline measures, 21 overlapping with the proportional hazard model [6], and an additional 5 which were prespecified in the statistical analysis plan [8]; two of which had similar properties to measures used in the proportional hazard model.

#### Decision tree classification:

We trained classification trees on 30 bootstraps of the augmented training and validation set (one on each bootstrap) to predict the primary composite outcome and provide confidence intervals. To prevent overfitting of the trees and check the stability of the results, we tuned basic parameters using cross-validation, such as the tree depth, and the minimum number of data points per leaf, and determined that a maximum of 6 leaves represented an optimal value. In other words, a typical decision tree model for this method has 6 terminal nodes representing 6 groups in the data. We then selected the decision tree with median test accuracy as our representative model to partition the set aside test data into 6 leaves with different distributions of outcome, creating subgroups for assessing HTE. Decision tree analysis was performed using the rpart library in R [9].

#### Random Forest classification:

We trained a random forest classifier by using 30 bootstraps of the augmented training and validation data to predict the primary composite outcome and obtain confidence intervals. Random forests, an ensemble model, are designed to reduce overfitting in the decision tree algorithm while maintaining its advantages [10]. We tuned the parameters using 10-fold cross-validation to prevent overfitting and check the stability of the results. The random forest models used 100 decision trees as base classifiers with each tree pruned to a maximum of 10 terminal nodes. The algorithm classified an instance by a majority vote across all the classification outputs of the individual decision trees. Votes were weighted by the individual predicted probabilities of the positive class before being aggregated. Unlike the decision tree, the leaf node groupings of a single decision tree in a random forest can no longer be used to identify subgroups. Therefore, using the classification probabilities (i.e., the probability of reaching an endpoint vs. not) as risk scores, the participants were stratified into subgroups by risk quintile, with group 1 containing the fifth with lowest predicted risk, and group 5 containing the fifth with highest predicted risk. Random forests were trained using the randomForest library in R [11].

### Assessment of model predictive ability

We used the proportional hazards model to predict risk of the composite outcome for US APREE participants in the testing set. The accuracy, sensitivity, specificity, and positive predictive value were computed at a risk prediction threshold of 50%. The area under the receiver operating characteristic curve (AUC ROC) was computed as the time dependent AUC at 5 years after randomization, in SAS 9.4 TS1M6 using proc phreg, participants’ predicted risk probabilities, and the nearest neighbor method. This procedure was repeated for the decision tree and random forest models. Calibration was assessed by comparing the mean predicted risk in each subgroup to the observed event rate in each subgroup. No formal tests were conducted to assess if significant differences existed between models. These metrics were used to assess the reliability of the subgroups generated by the models; but were not, in themselves, indicative of the model’s ability to reveal HTE.

### Model performance in heterogeneity of treatment effect analyses

We assessed HTE on the absolute scale by computing the 5-year absolute risk reduction imparted by aspirin. Starting with the groups developed with the extant proportional hazard model, disability free longevity at 5-years was computed using the Kaplan–Meier estimator for each combination of treatment and subgroup assignment. Then, the 5-year event rate was calculated as one minus the 5-year disability free longevity rate. Last, the 5-year absolute risk reduction (ARR) was computed as event rate in the group assigned to placebo minus the event rate in the group assigned to aspirin therapy, with the 95% confidence interval computed as defined in equation in Appendix 3. A meta-analysis was conducted to identify if ARR varied by subgroup. Cochran’s Q-test was interpreted to determine whether significant HTE was detected on the absolute scale. We assessed HTE on the relative scale by computing the hazard ratio for aspirin therapy in each subgroup. The Wald Chi-Squared test for the interaction of subgroup and treatment assignment was interpreted to determine whether significant HTE was detected on the relative scale. This procedure was repeated for the decision tree and random forest model.

## Results

### Participants

In total, 19,114 participants enrolled in the ASPREE study, 2,411 of whom were from the United States. A participant flow diagram is shown in Fig. 1. After excluding 120 participants due to missing data, 1,141 were assigned to the Training & Validation set, and 1,150 were assigned to the Testing set for a total of 2,291 participants analyzed after accounting for missing data. The baseline characteristics for the final study population set by treatment group are shown in Table 1.

**Table 1:** Participant descriptive statistics

		**Aspirin**		**Placebo**	
		***n***	**%**	***n***	**%**
Total		1136		1155	
**Demographics**					
Sex	Female	758	66.7%	767	66.4%
	Male	378	33.3%	388	33.6%
Residence	At home alone	418	36.8%	455	39.4%
	At home with family	708	62.3%	686	59.4%
	In residential home	10	0.9%	14	1.2%
Years of Education	< 9	47	4.1%	61	5.3%
	9–11	57	5.0%	60	5.2%
	12	247	21.7%	213	18.4%
	13–15	333	29.3%	332	28.7%
	16	200	17.6%	225	19.5%
	17–21	252	22.2%	264	22.9%
Race/Ethnicity	Hispanic	179	15.8%	168	14.6%
	Black	414	36.4%	426	36.9%
	White	520	45.8%	534	46.2%
	Other	23	2.0%	27	2.3%
**Prevalent Diagnoses & Drug Use**					
Diabetes	No	958	84.3%	955	82.7%
	Yes	178	15.7%	200	17.3%
Hypertension	No	338	29.8%	363	31.4%
	Yes	798	70.3%	792	68.6%
Dyslipidemia	No	598	52.6%	602	52.1%
	Yes	538	47.4%	553	47.9%
Personal History of Cancer	No / Unsure	933	82.1%	955	82.7%
	Yes	203	17.9%	200	17.3%
Previous Regular Aspirin Use	No	713	62.8%	737	63.8%
	Yes	423	37.2%	418	36.2%
**Risk Factors**					
Smoker Status	Current	71	6.3%	91	7.9%
	Former	457	40.2%	445	38.5%
	Never	608	53.5%	619	53.6%
Alcohol Use	Current	695	61.2%	699	60.5%
	Former	150	13.2%	157	13.6%
	Never	291	25.6%	299	25.9%
BMI	Underweight (BMI < 18.5)	6	0.5%	3	0.3%
	Normal (18.5 ≤ BMI < 25)	273	24.0%	284	24.6%
	Overweight (25 ≤ BMI < 30)	453	39.9%	471	40.8%
	Obese (30 ≤ BMI)	404	35.6%	397	34.4%
Frailty	Not frail	472	41.6%	425	36.8%
	Pre-frail	612	53.9%	674	58.4%
	Frail	52	4.6%	56	4.9%
Family History MI	No	693	61.0%	741	64.2%
	Yes	443	39.0%	414	35.8%
**Outcome**					
Endpoint Reached	No	1027	90.4%	1023	88.6%
	Yes	109	9.6%	132	11.4%
		**Aspirin**		**Placebo**	
		**Mean**	**St Dev**	**Mean**	**St Dev**
Age		73.9	5.4	73.8	5.5
**Lab**					
HDL Cholesterol (mmol/L)		1.6	0.5	1.6	0.5
LDL Cholesterol (mmol/L)		2.9	0.9	2.9	0.8
eGFR CKD Formula (mL/min/1.73m2)		74.1	16.3	75.7	16.5
Hemoglobin (g/dL)		13.7	1.3	13.7	1.3
**Physical measurements**					
Systolic Blood Pressure (mmHg)		134.9	17.4	135.4	16.9
Diastolic Blood Pressure (mmHg)		77.3	10.1	77.5	10.1
Abdominal Circumference (cm)		97.7	14.4	97.5	13.9
Mean Dominant Hand Grip Strength (kg)		25.1	11.5	25.3	11.9
Mean Gait Speed (seconds/3 meters)		3.8	1.6	3.8	1.8
**Cognitive function**					
Modified Mini-Mental State Examination (3MS)		93.3	5.1	93.7	5.0
Center for Epidemiological Studies – Depression Score (CES-D 10 questions)		3.3	3.2	3.5	3.6

### Model predictive ability

Model accuracy, sensitivity, specificity, positive predictive value, and area under the curve (AUC) are displayed in Table 2, and receiver operator curves (ROC) in Appendix 4. The accuracy and AUC in the proportional hazards model were 0.89 and 0.674 respectively. The decision tree model had a lower accuracy, but similar AUC (0.69, 0.672). The random forest model had a similar accuracy to the proportional hazards model, but higher AUC (0.88, 0.732). The sensitivity of the proportional hazards model was 0.12. Sensitivity was much greater in the decision tree model (0.64), but again, similar to the proportional hazards model in the random forest model (0.15). The positive predictive value of the proportional hazards model was 0.44. Positive predictive value was much lower in the decision tree model (0.20) and was again similar to the proportional hazards model in the random forest model (0.36). The predicted risk and observed risk were most similar in the proportional hazards model; however, the predicted risk was much greater than the observed values in the much greater than the observed values in the decision tree and random forest models, as show in Appendix 5.

**Table 2:** Predictive performance for having had the event by the end of study in the test set

Model	Accuracy	Sensitivity	Specificity	PPV	AUC
Proportional Hazards	0.89 (0.87–0.91)	0.12 (0.06–0.18)	0.98 (0.97–0.99)	0.44 (0.27–0.60)	0.674
Decision Tree	0.69 (0.67–0.72)	0.64 (0.55–0.72)	0.70 (0.67–0.73)	0.20 (0.16–0.24)	0.672
Random Forest	0.88 (0.86–0.90)	0.15 (0.09–0.21)	0.97 (0.96–0.98)	0.36 (0.24–0.51)	0.732

### Model performance in heterogeneity of treatment effect analyses

**Fig. 2:**
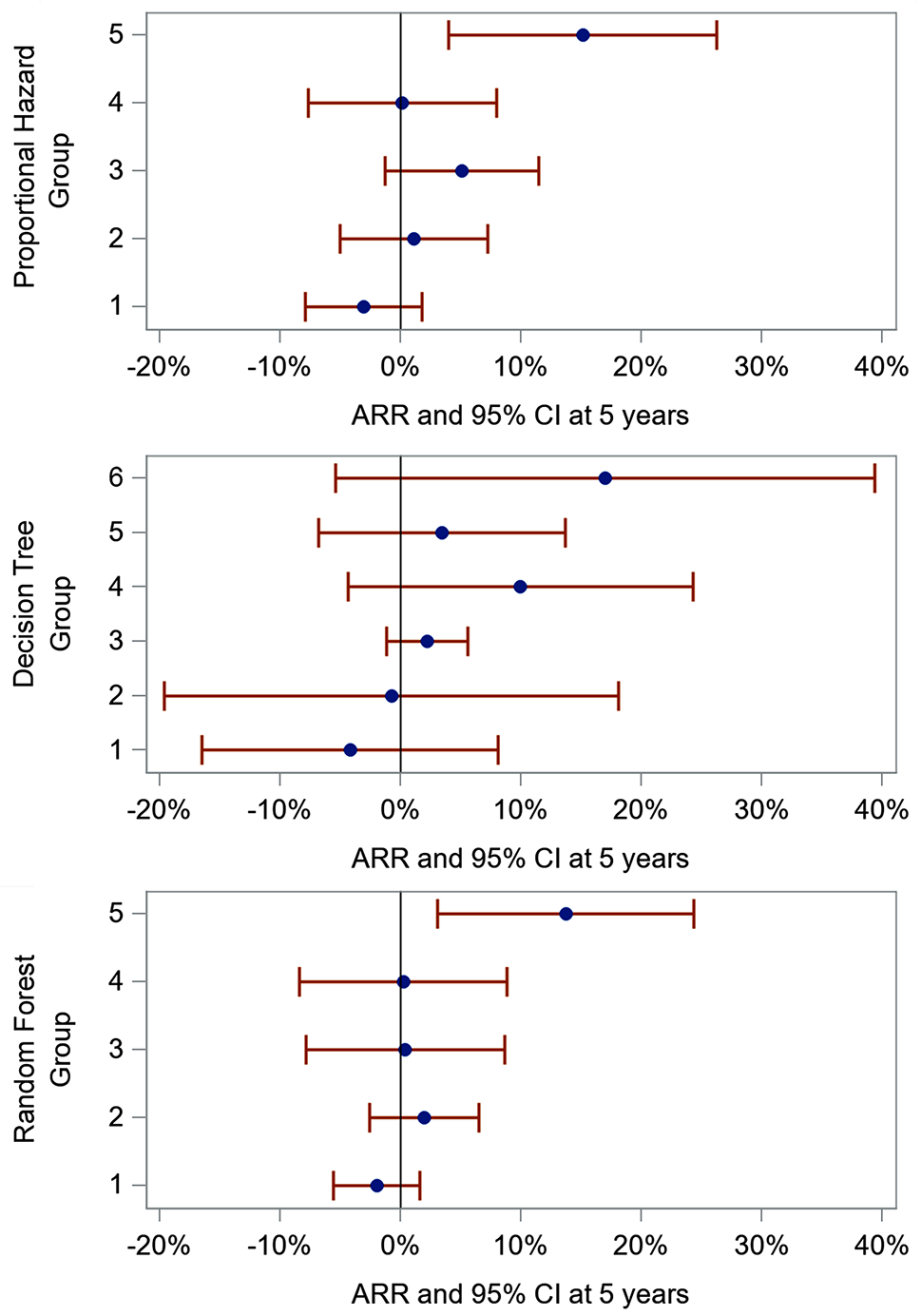
Absolute risk difference at 5 years by model, values reflect data in Appendix 6. *Each proportional hazard group and each random forest groups contained 230 participants. The Decision tree model contained 56 participants in group 1, 41 in group 2, 668 in group 3, 100 in group 4, 244 in group 5, and 41 in group r

Significant HTE was detected on the absolute scale in the proportional hazard model (*p* = 0.033), Appendix 6. The findings are shown graphically in Fig. 2. Using the proportional hazards model, participants in group 5 (the fifth with highest predicted risk) experienced significantly fewer events when on aspirin therapy compared to placebo (ARR = 15.1%; 95% CI 4.0–26.3%). Using the decision tree model, all subgroups had an absolute risk difference which included a difference of zero in the 95% confidence interval. Similar to the proportional hazard model, when using the random forest classifier, participants in group 5, experienced fewer events on the absolute scale when assigned to aspirin therapy compared to placebo (ARR = 13.7%; 95% CI 3.1–24.4%); however, the difference across groups was not significant (*p* = 0.085). The number needed to treat for group 5 in the proportional hazard model and random forest are 6.6 (3.8 to 25.1) and 7.3 (4.1 to 32.5) respectively. None of the models exhibited HTE on the relative scale (Wald Chi-Squared test for interaction subgroup-values ranged from 0.28 to 0.72), Appendix 6.

## Discussion

We investigated non-parametric approaches (supervised machine learning models) as compared to a standard, semi-parametric approach for creating subgroups. We then compared the models in their utilization in HTE models. Although externally developed outcomes risk models (models developed independently of the cohort they will be applied) are preferred in HTE analyses, internally developed prediction models are appropriate when high quality external models are not readily available [3]. To the best of our knowledge, non-parametric machine learning approaches have not been compared to the more widely utilized Cox proportional hazards model in terms of stratifying risk for discovery of potential treatment heterogeneity. To permit a more equal comparison between techniques, we limited our machine learning approaches to using candidate factors which had been commonly used in previous assessments of ASPREE (the measures indicated in Appendix 1). Our modeling implementations included participants in both treatment arms, not just the control arm, for model development, as recommended [3].

Our internally developed, non-parametric random forest algorithm performed similarly compared to a previously developed proportional hazards-based model in terms of both outcome discrimination and HTE identification. For both models, participants who were in the group with the highest predicted risk experienced fewer events on the absolute scale when treated with aspirin compared to placebo. However, the overall difference across groups was significant for only the proportional hazards model. Although confidence intervals were wide, at least in part a consequence of the limited number of participants in the subgroups, the point estimate for the absolute risk reduction was greater in participants with a higher predicted risk by the decision tree model.

Supervised machine learning models offer benefits over proportional hazard models as well as limitations. First, the supervised machine learning models subgroup based on baseline data while the proportional hazards model takes time into consideration, which adds complexity and an additional dynamic variable. Second, the supervised machine learning models make no assumptions on the distribution of the data while the semi-parametric proportional hazards model does. Third, there is less data pre-processing required for the supervised machine learning models than the proportional hazards model. Fourth, supervised machine learning models provide ranking of variables based on their ability to discriminate between research participants with and without outcome while coefficient magnitude is used as a proxy for feature importance in semi-parametric proportional hazards models. Fourth, supervised machine learning models overcome the potential of data leakage in HTE analyses as two Cox proportional hazards models with the same outcome are not used, while this situation occurs when a proportional hazards model for subgroup generation is utilized. However, supervised machine learning models used in these analyses only predict occurrence of outcome while the proportional hazards model predicts time-to-event.

The choice between the supervised learning models (random forests vs. decision tree) has benefits and limitations to consider. The random forest is designed to decrease the variability in decision tree and provide more stable predictions. Decision tree models are the most explainable as they can be directly translated into human understandable rules. Decision tree follows an “if-then” format where conditions on variables are evaluated in sequence to determine the final prediction.

A limitation of the study is the selected supervised machine learning models did not consider time-to-event capture. There are such models including survival trees and random survival forests that can account for time-to-event information and censorship more directly [12, 13]. Although recent work has shown poor agreements between them, there are other families of machine learning approaches that have been proposed to identify individualized treatment rules, such as causal forests [14]. In addition, the supervised learning models did not take censorship into account. However, we chose to first examine the more ubiquitous and understood methods of random forests and decision tree first. Strengths of this study include describing a process for comparing different outcome risk model methods for generating subgroups for HTE analyses. The key learning point of our work is that the choice of outcomes risk modelling to generate subgroups for HTE analyses is a balance of trade-offs that must be explicitly stated in the methods section of a manuscript. As other options become available for outcomes risk modelling, they will have to be compared and contrasted with existing methods to better appreciate the trade-offs. In addition, we highlight that utilizing multiple methods at outcome risk models may be beneficial in determining the robustness of HTE analysis results.

Potential future work includes comparing the feature importance characteristics of outcome prediction models for HTE analyses as this effort could identify mechanistic pathways to explain HTE analyses findings. Such work could result in further hypothesis generation for the tailored application of health interventions. Also, confirmation of these findings regarding the trade-offs of outcome risk model choices in HTE analyses in other mid-sized clinical trial derived datasets is needed.

## Conclusion

This study evaluated non-parametric machine learning models as risk predictors for HTE subgrouping and a previously developed proportional hazards model as a comparator. Non-parametric partition-based machine learning methods can generate internal subgroups for HTE analysis which exhibit similar performance to conventional regression-based approaches. Supervised machine learning models may be promising contenders for internally developed models for subgroups analysis when compared to a traditionally used, risk-based, semi-parametric model. They may produce comparable groupings based on outcomes risk but with less training data, less variables (omitting time and self-selecting important features), and less assumption on the underlying structure of the data.

### Electronic supplementary material

Below is the link to the electronic supplementary material.

Supplementary Material 1

## Data Availability

For access to the ASPirin in Reducing Events in the Elderly (ASPREE) project data, visit ams.aspree.org.Code for this project and final hyperparameters are available at: https://anonymous.4open.science/r/P428_HTE-781 A/README.md.
