# Supplementary material for: Outcome risk model development for heterogeneity of treatment effect analyses: a comparison of non-parametric machine learning methods and semi-parametric statistical methods
Source: BMC Med Res Methodol. 2024 Jul 23;24:158. doi: 10.1186/s12874-024-02265-8 (PMC11265457; doi:10.1186/s12874-024-02265-8)
Supplement: Supplementary file 1 — Supplementary Material 1 [file 12874_2024_2265_MOESM1_ESM.docx]

**Supplementary Material**

**Appendix 1: Candidate Predictors for Disability Free Survival**

**Appendix 2: Equations for Proportional Hazards Predictive Risk Model**

**Appendix 3: Equations for Absolute Risk Reduction**

**Appendix 4: Area under the Receiver Operating Characteristic Curves (AUC ROC)**

### **Appendix 5: Calibration Graph**

**Appendix 6: Aspirin Heterogeneity of Treatment Effect for Disability-Free Survival**

**Appendix 1: Candidate Predictors for Disability Free Survival**

| **Measure** | **Proportional Hazards *** | **Supervised Machine Learning Models** |
| --- | --- | --- |
| **Demographics** |  |  |
| Gender | ✓ | ✓ |
| Age | ✓ | ✓ |
| Living Status | ✓ | ✓ |
| Years of Education | ✓ | ✓ |
| Race/Ethnicity | ✓ | ✓ |
| **Prevalent Diagnoses** |  |  |
| Diabetes | ✓ | ✓ |
| Antihypertensive Agents | ✓ |  |
| Hypertension | - | ✓** |
| Lipid-Lowering Agents | ✓ | - |
| Dyslipidemia | - | ✓** |
| Personal history of Cancer | - | ✓** |
| **Risk Factors** |  |  |
| Smoking History | ✓ | ✓ |
| Alcohol Use | ✓ | ✓ |
| Frailty | - | ✓** |
| Family History of Myocardial Infarction | ✓ | ✓ |
| **Lab** |  |  |
| high-density lipoprotein cholesterol (HDL) | ✓ | ✓ |
| Low density lipoprotein cholesterol (LDL)*** | ✓ | ✓ |
| Estimated Glomerular Filtration Rate (eGFR) | ✓ | ✓ |
| Hemoglobin | ✓ | ✓ |
| **Physical measurements** |  |  |
| Systolic Blood Pressure | ✓ | ✓ |
| Diastolic Blood Pressure | ✓ | ✓ |
| Body mass index (BMI) | ✓ | ✓ |
| Abdominal Circumference | ✓ | ✓ |
| Mean Dominant-Hand Grip Strength | ✓ | ✓ |
| Mean Gait Speed | ✓ | ✓ |
| **Aspirin use** |  |  |
| Randomization to Aspirin | ✓ | - |
| Previous Regular Use of Aspirin | - | ✓** |
| **Cognitive function** |  |  |
| Modified Mini-Mental State Examination (3MS) | ✓ | ✓ |
| Depression score (Centre for Epidemiological Studies- Depression-10 questions) (CES-D) | ✓ | ✓ |

* Neumann JT, Thao LTP, Murray AM, et al. Prediction of disability-free survival in healthy older people. GeroScience. 2022;44(3):1641-1655. doi:10.1007/s11357-022-00547-x

**Measures used for prespecified subgroup analysis in the initial ASPREE publication on disability free survival: Wolfe R, Murray AM, Woods RL, Kirpach B, Gilbertson D, Shah RC, Nelson MR, Reid CM, Ernst ME, Lockery J, Donnan GA, Williamson J, McNeil JJ. The aspirin in reducing events in the elderly trial: Statistical analysis plan. Int J Stroke. 2018;13(3):335-338.

## **Appendix 2: Equations for *Proportional Hazards Predictive Risk Model [8].***

Model for women: 5-year risk (%) = [ 1-(4.787198 × 10^-8^ )^exp (LP)^ ] × 100%

LP = [ 0.0812863 × Age]

+ [ -0.0785434 × 3MS]

+ [ -2.1058784 × Gait speed]

+ [ 13.6499575 × (Gait speed-0.8391608)^3^] (If Gait speed > 0.8391608)

+ [-27.8419999 × (Gait speed-0.9852217)^3^] (If Gait speed > 0.9852217)

+ [ 14.1920424 × (Gait speed-1.1257036)^3^] (If Gait Speed > 1.1257036)

+ [ -0.0824954 × BMI]

+ [ 0.0022333 × (BMI-24.48889)^3^] (If BMI > 24.48889)

+ [ -0.0040281 × (BMI-27.46667)^3^] (If BMI > 27.46667)

+ [ 0.0017948 × (BMI-31.17188 )^3^] (If BMI > 31.17188)

+ [ 0.3885922 × (CES-D > 8) ]

+ [ -0.0188137 × Grip strength]

+ [ 0.2644989 × Diabetes]

Model for Men: 5-year risk (%) = [1-(5.304956 × 10^-8^)^exp (LP)^ ] × 100%

LP = [ 0.0791378 × Age]

+ [ -0.0614952 × 3MS]

+ [ -0.9872901 × Gait speed]

+ [ -0.0175855 × Grip strength]

+ [ -0.6700744 × (Smoking = Former/Never) ]

+ [ -0.0809386 × BMI]

+ [ 0.0034296 × (BMI-25.29407)^3^] (If BMI > 25.29407)

+ [ -0.0064518 × (BMI-27.55675)^3^] (If BMI > 27.55675)

+ [ 0.0030222 × (BMI-30.12438)^3^] (If BMI > 30.12438)

+ [ -0.0157298 × eGFR]

+ [ 0.0000532 × (eGFR-63.96903)^3^] (If eGFR > 63.96903)

+ [ -0.0001092 × (eGFR-74.24563)^3^] (If eGFR > 74.24563)

+ [ 0.0000560 × (eGFR-84.00224)^3^] (If eGFR > 84.00224)

**Appendix 3: Equations for Absolute Risk Reduction**

- CER: The control event rate, i.e., the event rate in the group assigned to placebo
- EER: The experimental event rate, i.e., the event rate in the group assigned to aspirin therapy
- ARR: Absolute risk reduction: CER-EER
- ARR 95% confidence interval: ARR ± (1.965$*$SE_ARR_)
- SE_ARR_: The Absolute Risk Reduction Standard Error: $\sqrt{\left( \frac{EER*(1-EER)}{n_{aspirin}} \right)+\left( \frac{CER*(1-EER)}{n_{placebo}} \right)}$
  - SAS Institute Inc. “Risks and Risk Differences”. SAS/STAT User’s Guide. 2019. documentation.sas.com/doc/en/pgmsascdc/9.4_3.4/statug/statug_freq_details54.htm

### **Appendix 4: Area under the Receiver Operating Characteristic Curve (AUC ROC)**

Proportional Hazard Model

AUC at 5 years = 0.674


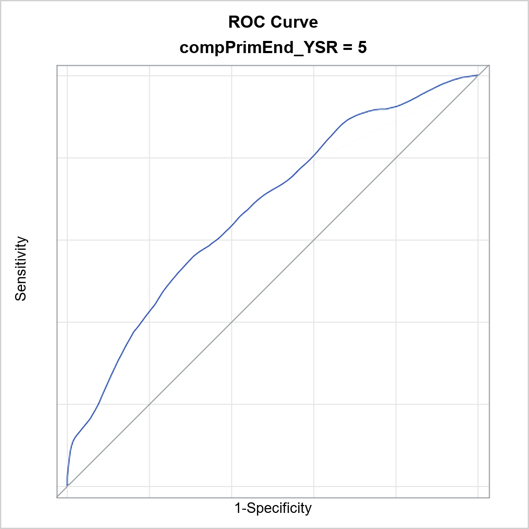


Decision Tree Model

AUC at 5 years = 0.672


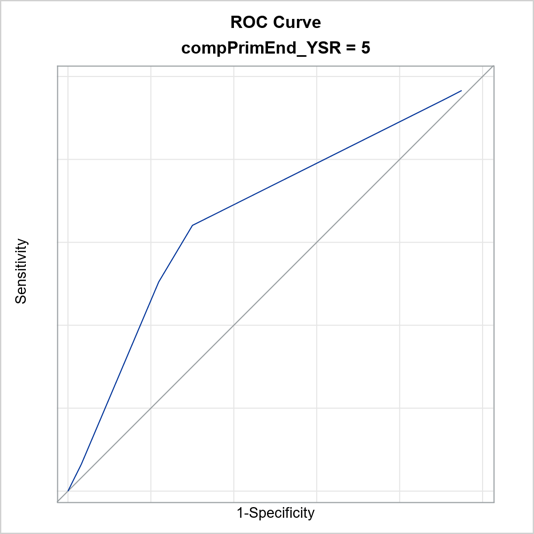


Random Forest Model

AUC at 5 years = 0.732


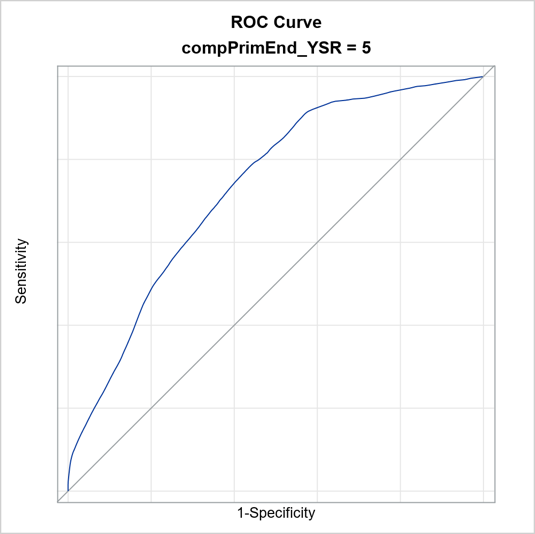


### **Appendix 5: Calibration Graph**

Proportional Hazard Model


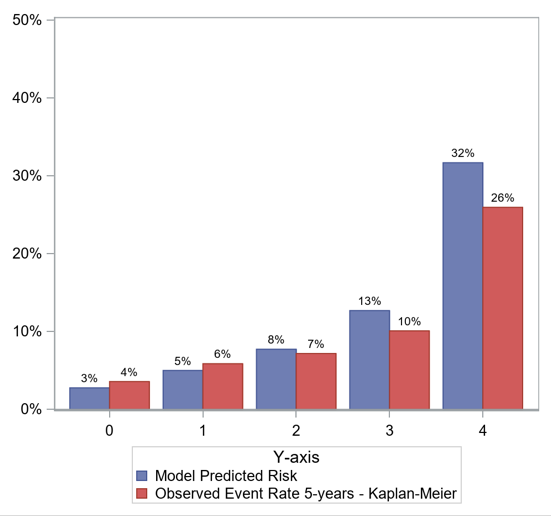


Decision Tree Model

69% 72% 77%


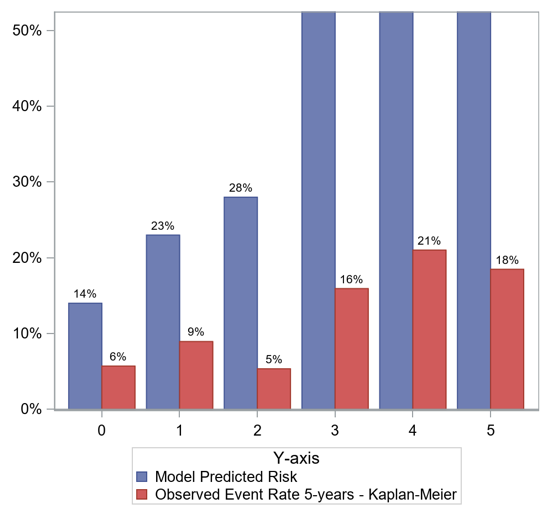


Random Forest Model


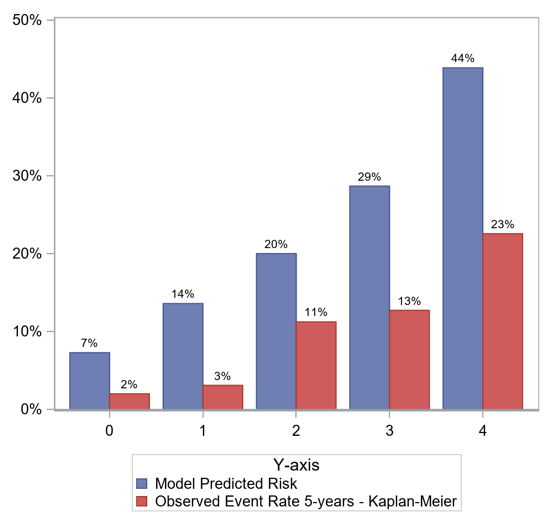


### **Appendix 6: Aspirin Heterogeneity of Treatment Effect for Disability-Free Survival**

|  | Events at 5 years / n per group (%) | | ARR at 5 years (95% CI) | HR (95% CI) |
| --- | --- | --- | --- | --- |
| Model | Aspirin | Placebo |  |  |
| Proportional Hazard |  |  | Cochran’s Q-test: p=0.033 | p-interaction: 0.280 |
| Group 1 | 5/111 (4.5%) | 2/119 (1.7%) | -3.05% (-7.89 to 1.78) | 1.83 (0.44 to 7.66) |
| Group 2 | 6/123 (4.9%) | 6/107 (5.6%) | 1.13% (-5.01 to 7.27) | 1.29 (0.46 to 3.63) |
| Group 3 | 3/101 (3.0%) | 11/129 (8.5%) | 5.08% (-1.31 to 11.47) | 0.42 (0.14 to 1.30) |
| Group 4 | 9/121 (7.4%) | 9/109 (8.3%) | 0.16% (-7.67 to 7.98) | 0.92 (0.41 to 2.04) |
| Group 5 | 17/112 (15.2%) | 31/118 (26.3%) | 15.14% (3.99 to 26.28) | 0.55 (0.33 to 0.93) |
| Decision Tree |  |  | Cochran’s Q-test: p=0.551 | p-interaction: 0.722 |
| Group 1 | 2/27 (7.4%) | 1/29 (3.4%) | -4.19% (-16.49 to 8.12) | 2.33 (0.21 to 25.72) |
| Group 2 | 1/29 (3.4%) | 1/12 (8.3%) | -0.76% (-19.62 to 18.11) | 0.38 (0.02 to 6.13) |
| Group 3 | 11/324 (3.4%) | 19/344 (5.5%) | 2.22% (-1.18 to 5.62) | 0.74 (0.39 to 1.40) |
| Group 4 | 5/51 (9.8%) | 8/49 (16.3%) | 9.96% (-4.35 to 24.27) | 0.84 (0.36 to 1.97) |
| Group 5 | 20/119 (16.8%) | 24/125 (19.2%) | 3.46% (-6.78 to 13.70) | 0.77 (0.44 to 1.34) |
| Group 6 | 1/18 (5.6%) | 6/23 (26.1%) | 17.00% (-5.39 to 39.38) | 0.18 (0.02 to 1.52) |
| Random Forest |  |  | Cochran’s Q-test: p=0.085 | p-interaction: 0.430 |
| Group 1 | 3/116 (2.6%) | 1/114 (0.9%) | -1.97% (-5.57% to 1.63%) | 2.97 (0.31 to 28.5) |
| Group 2 | 2/121 (1.7%) | 4/109 (3.7%) | 1.96% (-2.57% to 6.48%) | 0.43 (0.11 to 1.73) |
| Group 3 | 8/105 (7.6%) | 12/125 (9.6%) | 0.39% (-7.86% to 8.63%) | 1.10 (0.50 to 2.42) |
| Group 4 | 11/114 (9.6%) | 14/116 (12.1%) | 0.23% (-8.38% to 8.85%) | 0.87 (0.42 to 1.80) |
| Group 5 | 16/112 (14.3%) | 28/118 (23.7%) | 13.73% (3.07% to 24.38%) | 0.59 (0.34 to 1.02) |

- Hazard ratios indicate the hazard for aspirin therapy vs placebo for each subgroup
  - Values less than 1 indicate benefit from aspirin therapy
- p-interaction indicates the p-value for the interaction of treatment and subgroup for each model
- ARR computed as the difference in the Kaplan–Meier rate of disability free survival at 5 years after randomization
  - Values greater than 0 indicate benefit from aspirin therapy
